# The TGFB1 gene is associated with curve severity but not with the development of adolescent idiopathic scoliosis: a replication study in the Chinese population

**DOI:** 10.1186/s12891-016-0863-8

**Published:** 2016-01-13

**Authors:** Leilei Xu, Weixiang Sun, Xiaodong Qin, Yong Qiu, Zezhang Zhu

**Affiliations:** Department of Spine Surgery, The Affiliated Drum Tower Hospital of Nanjing University Medical School, Zhongshan Road 321, Nanjing, 210008 China

**Keywords:** Adolescent idiopathic scoliosis, TGFB1, Polymorphism, Replication

## Abstract

**Background:**

The transforming growth factor beta-1 (TGFB1) gene was recently reported to be a new susceptible gene of adolescent idiopathic scoliosis (AIS) in Russian population. This study aimed to replicate the relationship between the TGFB1 gene and the susceptibility of AIS in a Chinese population, and to further describe its association with the curve severity.

**Methods:**

A total of 1251 female AIS patients and 994 age-matched healthy controls were included in this study. The rs1800469 of TGFB1 gene was genotyped for all participants using the PCR-based Invader assay. The differences of genotype and allele distributions between AIS patients and healthy controls were assessed using the Chi-square test. One-way ANOVA test was used to compare the mean Cobb angles among patients with different genotypes.

**Results:**

There was no significant difference in terms of the genotype and the allele frequency between the patients and the controls. The mean Cobb angle was 34.7 ± 11.9° (range 25–61°). Case-only analysis showed that rs1800469 was significantly associated with the curve severity. Patients with genotype TT had remarkably higher curve magnitude (39.1 ± 12.8°) than those with genotype CT (34.8 ± 11.1°) or CC (32.1 ± 10.6°).

**Conclusions:**

The TGFB1 gene may not be a predisposition gene of AIS in the Chinese population. However, it can play a role in the curve progression of AIS. Replication studies in other ethnic groups are warranted to understand the implication of TGFB1 gene in AIS.

## Background

Adolescent idiopathic scoliosis is a structural curvature of the spine that affects millions of children in the world [1–3]. Although numerous studies have been performed to investigate the etiology of AIS, there was still a limited understanding of its pathogenesis [4–7]. As a complex multi-factorial disease, AIS was believed to be resulted from the interaction among multiple genetic loci as well as environmental factors [8, 9]. Through genetic linkage analysis, a few susceptible loci have been proposed to be closely related to AIS [10–12]. Subsequently, more predisposition genes of AIS were identified through association studies, including estrogen receptor 1 (ESR1) [13, 14], estrogen receptor 2 (ESR2) [15], matrilin 1 (MATN1) [16], melatonin receptor 1B (MTNR1B) [17], tryptophan hydroxylase 1 (TPH1) [18], interleukin-6 (IL-6) [19, 20], C17orf67 and DOT1L [21]. In recent years, the pathogenesis of AIS was further investigated through genome-wide association studies (GWASs). Sharma et al. [22] reported a strong association between the CHL1 gene and AIS in 419 white families. GWAS in Japanese population confirmed that the ladybird homeobox 1 (LBX1) gene and G protein—coupled receptor 126 (GPR126) gene were significantly associated with the development of AIS [23, 24]. Specifically, the LBX1 gene was confirmed as the susceptibility gene of AIS by a large-scale study in the European population [25].

Although the genetic background of AIS has been investigated extensively through genetic association study, spurious results may be produced along with those intriguing findings. As a well established AIS susceptibility gene in the Caucasian population, the CHL1 gene failed to be replicated in the Chinese Han population [26]. The ESR1 gene was firstly confirmed to be associated with AIS in the Japanese population and later successfully validated in the Chinese and the Caucasian population [13, 14, 27, 28]. Interestingly, this association was not supported by other 3 replication studies separately performed in the Asian and the Caucasian population [29–31]. Takahashi et al. [30, 32] failed to validate previously reported susceptible genes of AIS in the Japanese population, including ESR2, MTNR1B, TPH1 and MATN1. Collectively, there exists a significant divergence between the Asian and the Caucasian populations regarding the association of susceptible genes with the pathogenesis of AIS. Moreover, this divergence can even exist in the same population. Therefore, the large-scale replication study is warranted to validate previously reported susceptible genes of AIS.

Recently, Ryzhkov et al. [33] reported that the genetic polymorphism of the transforming growth factor beta-1 (TGFB1) gene was significantly associated with the onset of AIS as well as the curve severity in the Russian population. Interestingly, mutations in TGFB family genes can lead to syndromic forms of scoliosis such as Marfan and Loeys Dietz Syndrome [34, 35]. It is therefore possible that common variants of TGFB1 can be associated with AIS through similar pathway. To our knowledge, there is still a lack of knowledge concerning the association of TGFB1 gene with AIS in the Chinese population. The primary purpose of this study was to replicate the relationship between the TGFB1 gene and the susceptibility of AIS in the Chinese population, and to further describe its association with the curve severity.

## Methods

### Patients

Under the approval of Ethics Committee of The Affiliated Drum Tower Hospital of Nanjing University Medical School, this case-control study recruited 1251 female AIS patients and 994 age-matched healthy female subjects who underwent the routine examination prior to the college admission. All the patients were diagnosed as AIS through clinical and radiological examinations during their visits to our center for the treatment. Patients were excluded from this study if having scoliosis secondary to known etiology, including congenital scoliosis, neuromuscular scoliosis, scoliosis secondary to skeletal dysplasia, and connective tissue abnormalities. The healthy subjects were confirmed through Adam’s Forward Bend Test by an experienced spinal surgeon (Q.Y.). All participants were from Chinese Han population living along Yangtze River.

To investigate the association between the target SNP and the curve severity, a subgroup of patients were included in the case-only study according to the following inclusion criteria: 1. having no history of brace treatment or any other conservative treatment for scoliosis; 2. either reaching skeletal maturity or undergoing fusion surgery due to curve progression. Patients were considered to have reached skeletal maturity if aged more than 16 years or having a Risser sign of 5. The curve severity was recorded as the Cobb angle measured at the latest visit after skeletal maturity for non-operated patients, or at the last visit before surgery for those undergoing surgery. Patients with Cobb angle more than 40° were assigned to the progressive curve group, and those with Cobb angle between 20 and 30° were assigned to non-progressive curve group. To investigate the association between the target SNP and the age of onset of AIS, all patients and their parents were queried on the chronological age when they initially noticed the deformity, which was recorded as the age of onset for each patient if accurately provided.

### Genotyping of the target SNP

Blood samples were collected for DNA analysis, with informed consent obtained from the participants. Genomic DNA was extracted from blood leukocytes by standard protocols (Qiagen K.K., Tokyo, Japan). The rs1800469 of the TGFB1 gene was genotyped using TaqMan SNP Genotyping Assay according to the manufacturer’s instructions, which was read with an ABI PRISM 7900HT sequence detection system (Applied Biosystems, Foster City, CA). Samples with ambiguous results were collected in the 96-well plate and later genotyped for precise results. Twenty percent of the samples were selected randomly to validate the reproducibility of the genotyping results.

### Statistical analysis

The Hardy-Weinberg equilibrium (HWE) test was used for both the patient and the control group. The differences of genotype and allele distributions between AIS patients and healthy controls were assessed using the Chi-square test. In addition, the Chi-square test was also used to compare the allele frequency between patients with progressive curve and those with non-progressive curve. The odds ratio (OR) was calculated using the minor allele as a reference. The One-way ANOVA test was used to compare the mean Cobb angles of different genotypes in case-only analyses. Similarly, the age of onset was also compared among different genotypes using the One-way ANOVA test. The SPSS software (version 17.0, Chicago, IL) was used for statistical analyses. Statistical significance was set at a *p* value of less than 0.05.

## Results

### Case-control association analysis

The rs1800469 was successfully genotyped for all patients and controls. The concordance rate was 100 % for samples genotyped in duplicate. No significant difference of genotype frequencies from the HWE test was noted in the patients (*p* = 0.13) or the normal controls (*p* = 0.10). As shown in Table 1, neither the genotype nor the allele frequency of the rs1800469 was significantly different between the patients and the controls.

**Table 1:** Results of association analysis for the rs1800469 in 1251 cases and 994 controls

	Patients (*n* = 1251)	Controls (*n* = 994)	*p* value	Odds Ratio ^a^ (95 % CI)
Genotype			0.92	N/A
CC	592	479		
CT	520	406		
TT	139	109		
Alleles			0.72	1.02 (0.93–1.11)
C	1704	1364		
T	798	624		

*N/A* indicates not available

^a^ Odds ratio was calculated with the minor allele T as reference allele

### Case-only association analysis

According to the inclusion criteria for the case-only association analysis, a subgroup of 746 patients were investigated to determine the association of the rs1800469 with the curve severity, among whom 349 had received fusion surgery, and the other 397 had been observed until skeletal maturity. The mean Cobb angle was 34.7 ± 11.9° (range 25–61°). As shown in Table 2, the rs1800469 was found to be associated with the curve severity. Patients with genotype TT had remarkably higher curve magnitude (39.1 ± 12.8°) than those with genotype CT (34.8 ± 11.1°) or CC (32.1 ± 10.6°). Moreover, the progressive curve group was found to have significantly higher frequency of allele T than the non-progressive curve group (34.2 % vs. 28.5 %, *p* < 0.05).

**Table 2:** Results of case-only analysis of the curve severity

Genotype	Number	Curve severity (degrees)	*p* value
CC	347	32.1 ± 10.6	<0.01
CT	314	34.8 ± 11.1	
TT	85	39.1 ± 12.8	

Another subgroup of 535 patients were investigated to determine the association of the rs1800469 with the age of onset of AIS. The mean age of onset was 11.4 ± 1.8 years (range 10.3–14.4 years). As shown in Table 3, no significant association was found between the rs1800469 and the age of onset.

**Table 3:** Results of case-only analysis of the age of onset

Genotype	Number	Age of onset (years)	*p* value
CC	263	11.2 ± 1.3	0.78
CT	217	11.7 ± 1.2	
TT	55	11.6 ± 1.4	

## Discussion

Genetic association study is a widely applied strategy to determine the genetic variants associated with AIS [13, 15–18, 21]. The most important step of genetic association study is to target the putative candidate gene that could be implicated in the pathogenesis of the disease. The TGFB1 is a multifunctional cytokine that could affect a variety of physiological processes including cellular proliferation, differentiation and the formation and degradation of extracellular matrix proteins [36]. Previous studies showed a significant difference in TGFB1 expression level between convex and concave side of AIS patients [37]. Moreover, the TGFB1 was found to play a role in the degeneration of intervertebral discs [38]. Herein, the TGFB1 gene could be a highly putative predisposition gene of AIS. In a recent report of Ryzhkov et al. [33], the TGFB1 gene was identified as a new susceptible gene of AIS. In addition, it could also be a modifier gene of AIS, since it was found to be related with the age of onset and the curve severity [33]. However, the role of TGFB1 as a predisposition gene of AIS has not been addressed in the Chinese population. A genetic replication study involving both patients and controls is required to address this issue.

Our results showed that the previously reported association of the rs1800469 with the susceptibility of AIS in the Russian population could not be replicated in the Chinese population. It is a common phenomenon to have a negative replication outcome of variants associated with complex traits. To be noted, there is actually a lack of consensus on most susceptible genes of AIS except for the LBX1 gene [26, 29, 30, 32, 39, 40]. The discrepancy may be resulted from the ethnic differences between the different populations or from a lack of statistical power. In the study of Ryzhkov et al. [33], only 300 cases and 300 controls were recruited for the association analysis. Moreover, the genotype frequency of the rs1800469 in the patients did not follow HWE test, which could hamper the reliability of their conclusion [33]. In this study, we included a cohort of patients and controls with much larger sample size to detect the effect sizes as shown in the study of Ryzhkov et al. [33]. Therefore, it was unlikely for the current replication to have false-negative association due to a lack of statistical power. Collectively, our data did not support the clinical application of this gene as a predisposition gene of AIS in the Chinese population.

Relationships between the TGFB1 gene and the curve severity and age of onset of AIS were also analyzed in the current study. We found that allele T and genotype TT of the rs1800469 was significantly associated with progressive curve of AIS. Previous studies have reported that allele T and genotype TT of the rs1800469 were associated with increased plasma levels of TGFB1 [41, 42]. Besides, an abnormally higher expression of TGFB1 in articular process cartilages was detected at the concave side of AIS as compared with the convex side [43]. Therefore, we speculated that the different expression of TGFB1 between the concave and convex side of AIS could be implicated in the progression of the curve. Although the TGFB1 gene was reported to be significantly associated with the age of onset [33], such association was not replicated in our analysis. Collectively, it is likely that the TGFB1 gene could be a modifier gene that plays a role in the curve progression of AIS. The functional variants of TGFB1 can be potentially applied to the precise prediction of curve progression in AIS patients and used to guide bracing or surgical treatment. Herein, functional analysis concerning the expression of TGFB1 gene in patients with progressive curve and non-progressive curve is warranted to explain its relationship with the curve progression.

## Conclusion

Considering the large sample size of the present study and strict inclusion criteria for the case-only analysis, we conclude that the TGFB1 gene may not be a predisposition gene of AIS in the Chinese population. However, the TGFB1 gene is likely to be implicated in the curve progression. Replication studies in other ethnic groups are warranted to understand the implication of the TGFB1 gene in AIS.

## Abbreviations

AIS: adolescent idiopathic scoliosis
ESR1: estrogen receptor 1
ESR2: estrogen receptor 2
GPR126: G protein-coupled receptor 126
GWAS: genome-wide association studies
IL-6: interleukin-6
LBX1: ladybird homeobox 1
MATN1: matrilin 1
MTNR1B: melatonin receptor 1B
OR: odds ratio
SNPs: single nucleotide polymorphisms
TGFB1: transforming growth factor beta-1
TPH1: tryptophan hydroxylase 1
